# The Genetic Basis of Tomato Aroma

**DOI:** 10.3390/genes12020226

**Published:** 2021-02-04

**Authors:** Matteo Martina, Yury Tikunov, Ezio Portis, Arnaud G. Bovy

**Affiliations:** 1 DISAFA, Plant Genetics and Breeding, University of Turin, 10095 Grugliasco, Italy; matteo.martina@unito.it; 2 Plant Breeding, Wageningen University & Research, P.O. Box 386, 6700 AJ Wageningen, The Netherlands; yury.tikunov@wur.nl

**Keywords:** tomato, *S. lycopersicum*, aroma, volatiles, QTLs

## Abstract

Tomato (*Solanum lycopersicum* L.) aroma is determined by the interaction of volatile compounds (VOCs) released by the tomato fruits with receptors in the nose, leading to a sensorial impression, such as “sweet”, “smoky”, or “fruity” aroma. Of the more than 400 VOCs released by tomato fruits, 21 have been reported as main contributors to the perceived tomato aroma. These VOCs can be grouped in five clusters, according to their biosynthetic origins. In the last decades, a vast array of scientific studies has investigated the genetic component of tomato aroma in modern tomato cultivars and their relatives. In this paper we aim to collect, compare, integrate and summarize the available literature on flavour-related QTLs in tomato. Three hundred and fifty nine (359) QTLs associated with tomato fruit VOCs were physically mapped on the genome and investigated for the presence of potential candidate genes. This review makes it possible to (i) pinpoint potential donors described in literature for specific traits, (ii) highlight important QTL regions by combining information from different populations, and (iii) pinpoint potential candidate genes. This overview aims to be a valuable resource for researchers aiming to elucidate the genetics underlying tomato flavour and for breeders who aim to improve tomato aroma.

## 1. Introduction

Tomato (*Solanum lycopersicum* L.) is one of the most important crops on the market, used worldwide as basis in many national traditional dishes [1]. Conventional wisdom suggests that breeding tends to reduce the genetic basis of a cultivated species, but tomato genetic diversity appears to have actually been enhanced in the last fifty years. A recent study [2] investigated the genetic diversity of cultivated tomato varieties in The Netherlands (NL) from 1950s to 2010s, observing that tomato commercial varieties from 1950s and 1960s were mainly homozygous, with narrow genetic variation among them. From 1970s onwards, genetic diversity in tomato has increased, thanks to the application of introgression breeding programs using wild relatives of tomato. The first genetic diversity boost appeared to take place with the introgression of tomato mosaic virus (ToMV), southern root-knot nematode (*Meloidogyne incognita*) and leaf mold disease (*Cladosporium fulvum*) resistance from *Solanum peruvianum* and *Solanum pimpinellifolium* [3,4,5]. These introgressions varied in size from ~5% of the chromosome (introgression of Cf-4 and Cf-9 on top of Chr.01 for resistance to *Cladosporium fulvum*) up to half a chromosome 9 (ToMV), and together led to a significant increase in overall genetic diversity [2]. A second diversity boost, starting in the late 1980s affected both fruit size and quality traits: in particular the introgression of parts of Chr. 4, 5 and 12 from *S. pimpinellifolium* led to fruit size variation among cultivated varieties and the introduction of cherry, cocktail and large fruited varieties to the NL market [3].

Not only disease resistance and fruit size, but also flavour has been targeted by breeding programs in the last thirty years [2]. The main components of tomato fruit flavour are: (i) sweetness, mainly determined by sugars; (ii) acidity, determined by the presence of organic acids; (iii) textural attributes, such as firmness and juiciness, and (v) aroma [6]. Tomato aroma is determined by the interaction of volatile compounds released by the tomato fruits with receptors in our nose, leading to a sensorial impression, such as “sweet”, “smoky”, or “fruity” aroma. More than 400 volatile compounds [7] have been detected in ripe tomato fruit and they are derived from primary metabolites, such as fatty acids and amino acids or secondary metabolites, such as phenylpropanoids and carotenoids. Only a fraction of these volatiles has been associated with specific human sensorial attributes or were shown to be present at concentrations above the so-called odor threshold. From an evolutionary point, these molecules evolved to attract seed dispersers, including animals and men. For humans they have a great economic impact, since they are important determinants of food quality and consumer preference [8,9]. Among them, about 20 compounds have been identified as the most contributing ones, based on their concentrations in fruits and their individual odor thresholds [10]. However, it has to be mentioned that the perception of aroma is determined by both the odor activity of individual VOCs and by interactions between them or with other non-volatile chemicals [11]. For instance, the presence of sugars or organic acids alters the perception of aromatic descriptors of samples with the same concentration of volatiles [12,13], while the perception of basic tastes, e.g., sourness or sweetness, can be modified by variation in VOCs accumulation [8,14,15]. Tomato aroma is therefore a hard-to-define trait and efforts have been made to develop prediction models for its different components [8,12,16,17]. This led to the identification of the main 21 VOCs impacting consumer liking (Table 1). Each of these compounds has its own characteristic odor, as retrieved from the Good Scents Company database [18]. The compounds can be grouped in five clusters, according to their biosynthetic origins [11]: (1) fatty acids-derived VOCs; (2) sulphur-containing and branched chain amino acids-derived VOCs; (3) carotenoid catabolism by-product phenolic VOCs; (4) phenolic VOCs; and (5) phenylpropanoid VOCs [19,20,21,22,23,24,25].

Breeders and breeding researchers aim to elucidate the genetic basis underlying important agronomic traits. This requires three essential elements: (1) genetic variation, (2) phenotypic variation and (3) methods to find associations between the genetic and phenotypic data. As outlined in more detail below, advances in the development of high-throughput (HTP) molecular marker platforms, the availability of genomic information and progress in phenotyping methodologies, such as metabolomics, have led to an increasing importance and use of marker-assisted breeding strategies, such as QTL analysis, association mapping and genomic prediction in current plant breeding practice and transformed plant breeding into a hight-tech industry.

*Marker-assisted introgression breeding*: Tomato is highly autogamous, a characteristic that, together with the loss of many genes and alleles during domestication and crop improvement, led to a narrow genetic basis of cultivated tomato, compared with its 12 wild relative species [26]. For this reason, wild relatives have been used as potential sources of lost alleles in the development of new cultivated tomato varieties [27,28]. Marker-assisted selection in plant breeding programs relies on genetic linkage analyses, which are based on the principle of genetic recombination during meiosis. This allows the construction of linkage maps, composed of genetic markers linked to genes or Quantitative Trait Loci (QTL’s) affecting traits of interest for a specific population. QTL analysis is mostly done using biparental segregating populations based on a cross of two contrasting genotypes. In order to discover and elucidate the genetic basis of agricultural traits, segregating populations have not only been made from intraspecific crosses, but also from interspecific crosses with various tomato wild relatives (*S. pimpinellifolium*, *S. pennellii, S. lycopersicoides* and *S. habrochaites*) in which genomic regions of the wild donor have been introgressed in the cultivated tomato genetic background, allowing the identification of potential new alleles for traits of interest [29,30,31,32,33]. Interestingly, researchers not only investigated *Solanum lycopersicum* wild relatives, but also *Solanum lycopersicum* var. cerasiforme L, the expected ancestor of the domesticated tomato [34].

*The impact of genomics*: In the last decades, the advances in genomics have provided new tools for discovering and tagging novel alleles and genes. The advent of next generation sequencing (NGS) techniques has considerably accelerated and simplified the genome-wide detection of single-nucleotide polymorphisms (SNPs), which have become the most popular molecular markers. The development of the reference tomato genome from the inbred cultivar “Heinz 1706” [35] represented a milestone in the genomic era. The comparison of the cultivated tomato genome with the genome of a wild relative *S. pimpinellifolium* revealed the potential of high-throughput sequencing in comparative genetics, confirming the previously reported introgression of *S. pimpinellifolium* in the “Heinz 1706” genome [36] and the identification of thousands of SNPs between the two relatives. Further genome and transcriptome resequencing aimed at detecting genetic variation in tomato paved the way for the development of relatively universal genotyping platforms (i.e., SNP arrays) that can be applied for the genetic analyses of different populations—the SolCap SNParray [37,38] and the CBSG array [3]. A further progress in the application of NGS is represented by the genotyping-by-sequencing (GBS) approaches, based on the use of restriction enzymes to decrease genome complexity before sequencing. These techniques include over a dozen of reduced-representation sequencing (RRS) approaches [39] and have been recently applied for high-resolution QTL mapping in tomato, especially in interspecific crosses [40,41,42,43]. These protocols have been applied in the development of high-density genetic maps in many different species [44,45,46,47,48,49,50,51,52,53,54,55], making it possible to perform comparative and quantitative genetics in virtually any genomic background. The current advances in genome sequencing technologies, such as a revolutionary increase of sequencing throughput and concomitant reduction in costs per sequenced nucleotide, allowed to unravel the genetic variation in tomato to its full extent [25,56,57] and has helped to realize that the concept of one or a few reference genomes is not sufficient to fully understand the genetic control of traits. For this reason, the pangenome concept has been introduced in plant genomics, investigating the sum of genes that can be found in a specific species [58,59,60,61,62,63]. Specifically, the pangenome is defined as “the full complement of genes of a species, which can be partitioned into a set of core genes that are shared by all individuals and a set of dispensable genes that are partially shared or individual specific” [64]. In the Solanaceae family, the pangenomes of tomato and pepper (*Capsicum* spp.) have been recently released, identifying missing genes involved in resistance mechanisms and quality traits [26,65,66], but eggplant (*Solanum melongena* L.) and potato (*Solanum tuberosum* L.) are still pangenome-orphan species. The availability of the tomato pangenome allowed the identification of presence/absence variations (PAVs) and the identification of structural variants (SVs) of functionally important genes [26,65]. Interestingly, [26] identified a rare promoter allele for the TomLoxC gene, a lipoxygenase that has been reported to be crucial in C5 and C6 lipid-derived volatiles biosynthesis and apocarotenoid production [67,68]. Moreover, the newly developed tomato pangenome [65] resolved the genomic region flanking *NSGT1*, a functional gene associated with the production of guaiacol, methylsalicylate and eugenol, three phenylalanine-derived volatiles. Five different haplotypes were identified in the analyzed tomato germplasm, providing new insights in the understanding of the genetic variation of *NSGT1*. Pangenome appears to be the most novel tool that breeders and researchers have at their disposal: it may facilitate the mining of natural genetic variation and could contribute to crop improvement by supporting molecular breeding programs and gene function studies.

*Linking genetic markers to traits*: In the genomics era, novel genotyping techniques and the availability of multitudes of molecular markers, in combination with new high-throughput phenotyping technologies (i.e., phenotyping platforms), supported the development of new methodologies to link genetic markers to phenotypic traits. Not only the above-mentioned QTL mapping has become more precise, also association mapping and genomic prediction are now widely used in breeding [69,70,71,72]. Genome-Wide Association Studies (GWASs) are based on genotyping of a set of accessions representing the variability in a given species and rely on the linkage disequilibrium (LD) between a marker and its associated trait [73]. This technique has been applied in tomato, identifying interesting associations for many fruit quality traits [74,75,76,77,78,79]. Zhao et al. performed a meta-GWAS analysis, by combining datasets of several GWAS panels. This analysis not only led to confirmation of existing, but also to the discovery of novel QTLs and candidate genes for several flavour-related traits [77]. Genomic Prediction (GP) is a selection tool that makes use of genetic markers to predict the genetic potential of untested lines in breeding [80]. While QTL mapping and GWAS rely on the (statistically-significant) association between phenotypic variation and specific molecular markers, GP calculates the genetic potential of breeding candidates by the application of Bayesian or mixed statistical models that take all the genome-wide marker information into account to predict the phenotype. Genomic prediction is a selection tool rather than a research tool and performs better with traits that are controlled by a large number of small-effect QTLs which are hard to detect by QTL analysis of mapping populations or GWAS [81,82]. Genomic prediction is particularly useful for traits for which phenotyping is expensive, difficult or time consuming, since no phenotyping is needed for selection, once a good prediction model based on data of a representative training population is available. This technique has been widely applied in animal selection [83,84,85], while its practical application in plant breeding is still limited to major crops, such as maize and wheat [82,86,87], in which QTLs for important traits, such as yield, have already been fixed in the elite germplasm [88], or to tree crops, where early selection is very useful and cost-effective [89].

The combination of all the above-mentioned approaches, except GP, led to identification of a multitude of QTLs for many agronomic and quality traits. In this review we aim to collect, compare, integrate and summarize the available literature on flavour-related QTLs in tomato. We selected 16 scientific papers and supplemental data focusing on QTLs for tomato aroma and fruit quality. This not only provides an overview of the known flavour QTLs, but the combined and integrated information also makes it possible to (i) pinpoint potential donors described in literature for specific traits, (ii) highlight important QTL regions by combining information from different populations, and (iii) pinpoint potential candidate genes. This overview aims to be a valuable resource for researchers aiming to elucidate the genetics underlying tomato flavour and for breeders who aim to improve tomato aroma.

## 2. Construction of a Unified QTL Map of Tomato Aroma

A literature search was performed with the aim of collecting articles reporting QTLs for tomato aroma. In order to compare and integrate QTLs from different studies, the availability of marker information was an essential requirement for inclusion in this review. The identified QTLs have been organized and are available as Supplemental Material (Table S5), reporting the biosynthetic pathway, the QTLs Genomic Regions (QGR), the QTL’s original name, the related compound, the chromosome, the correlated markers, their position in cM and bp, their *p*-value, their LOD score, the SolycID of the gene in which the markers have been found, the percentage of explained variation by the QTL, the effect, the donor parent, the crossing population or association panel used and the reference of the primary resource.

We identified 16 articles reporting QTLs for tomato aroma including marker information (Table 2). In the pre-genomics era only genetic linkage positions (cM) of QTLs could be reported, since a reference genome sequence was not available the. This made it difficult to align QTLs of that period with the more recent studies utilizing modern genomics technologies. To circumvent this, the physical position of genetic markers was retrieved from the tomato genome (https://solgenomics.net/search/markers (accessed on 20 November 2020)) whenever marker sequence information was available, using the SL2.50 genome version.

**Figure 1:**
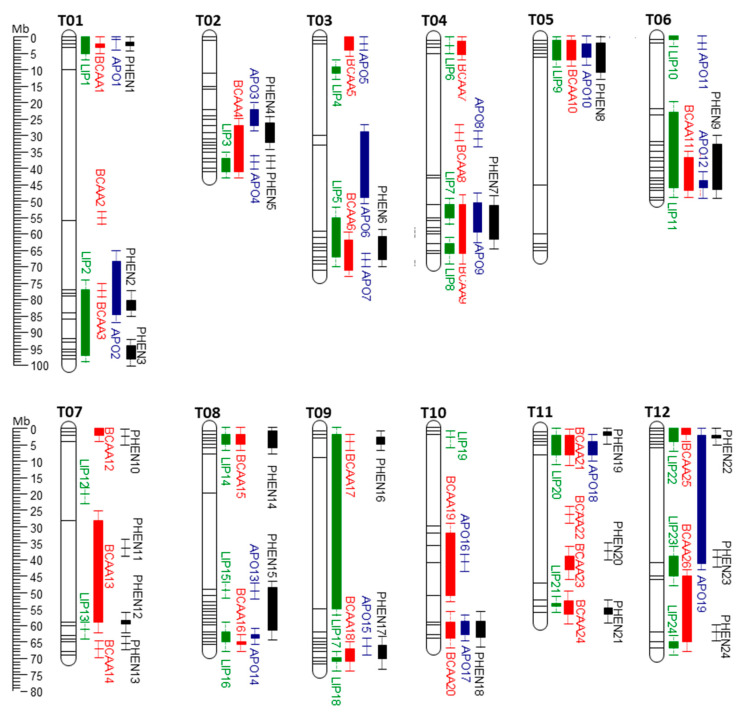
VOCs QGRs chromosome map (T01–T12) obtained from the reported literature. Green: Lipid VOCs QGRs; Red: BCAA VOCs’ QGRs; Blue: Apocarotenoid VOCs QGRs; Black: phenolic VOCs QGRs. The estimated confidence interval (±2.5 Mb), has been reported in the figure.

Two criteria were used to cluster QTL information from the different studies into QTLs Genomic Regions (QGRs) in a unified physical map (Figure 1): (i) a biochemical relationship between aroma volatiles and their possible precursors, such as similar chemical structure or common biosynthetic pathway and (ii) overlapping of the QTLs. Since most of the studies only reported the most significant marker(s) of an identified QTL, while information on genetic confidence intervals or (average) linkage disequilibrium (LD) decay was lacking, it was virtually impossible to determine the size of QTL regions. For this reason, we standardized the potential positional error across the reported studies by setting an empirically defined window of ±2.5 Mb around each identified QTL. This window was derived from the average inter-study standard deviation of QTL positions of the three most functionally explored tomato aroma loci—floral aroma on chromosome 4 [25,90,99,102], smoky aroma on chromosome 9 [25,90,102] and the malodorous locus on chromosome 8 [92,93,99]. The major genetic factors underlying these QTLs were identified and, therefore, the dispersion of QTL positions in these different studies could most likely be attributed to non-genetic sources of variation. This window as well as resulting QGRs only serve as means to classify QTLs and indicate the possibility that the individual QTLs they harbor may be affected by one or a few co-localized genetic factors.

The identified QGRs were mined for the presence of candidate genes based on their annotation (ITAG2.40) and on their expression in tomato fruit, using publicly available dataset and tools [103]. Candidate genes were defined by two criteria: (1) genes belonging to families known from literature to be involved in VOCs biosynthesis and expressed in tomato fruit and (2) genes reported in literature with a demonstrated function in VOCs biosynthesis, irrespective of their expression in tomato fruit. A complete list of the potential candidate genes (with and without expression in the fruit tissues) can be found as Supplemental Material (Tables S1–S4). The genes which have been demonstrated to functionally underlie aroma QTLs in tomato were highlighted in bold.

## 3. Fatty Acids Derived Volatiles (FA VOCs)

The volatile compounds originating from the degradation of linolenic and linoleic acid accumulate during tomato ripening and can also markedly increase their emission upon fruit tissue disruption. They provide a note of freshly cut grass to the aroma bouquet [104]. These compounds are the most abundant volatiles in tomato fruit and are mainly represented by the C_5_ volatile 1-penten-3-one, a few C_6_ volatiles, such as 1-hexanol, (*Z*)-3-hexenal, (*E*)-2-hexenal and hexanal, the C_7_ volatile (*E*)-2-heptenal and the C_10_ VOC (*E*,*E*)-2,4-decadienal [8,11,16,17]. Although their high accumulation in ripe fruits may suggest that these compounds are very important determinants of tomato flavour, some studies provide evidence that impact of their quantitative variation on consumer liking may be limited [8,67], likely due to their in general high abundance and low odor thresholds, e.g., for (*Z*)-3-hexenal these values are 12,000 nLּL^−1^ and 0.25 nLּL^−1^, respectively [21].

### 3.1. Biosynthesis of FA VOCs

**Figure 2:**
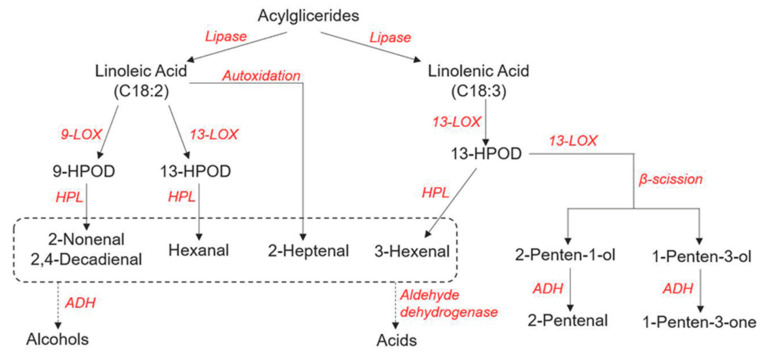
Overview of the lipid VOCs pathway, adapted from [118,119,120].

During tomato fruit ripening, free fatty acids, mainly linolenic and linoleic acid, are derived from the catabolism of acylglycerides from disintegrating cellular membranes, by the action of lipases [87,88]. Linolenic and linoleic acid can be further catabolized by means of β-oxidation, α-oxidation, or the lipoxygenase pathway [68,105,106,107]. In tomato fruit the latter is the most important for the production of volatiles, which occurs through two steps: (i) fatty acids are deoxygenated by means of lipoxygenases (LOX), which are classified as 13-LOX and 9-LOX and are leading respectively to 13-hydroperoxides and 9-hydroperoxides [67,108]; (ii) hydroperoxides are catabolized by means of hydroperoxide lyases (HPL), also classified as 13-HPL and 9-HPL, leading to an oxoacid and a volatile aldehyde. Volatile aldehydes can be converted into alcohols by means of alcohol dehydrogenases (ADH; [109,110,111,112,113,114]). According to the literature ([68]), 13-LOX enzymes are mainly involved in the synthesis of (*Z*)-3-hexenal from linolenic acid and hexanal from linoleic acid. Among these enzymes, TomloxC has shown significant correlation with the production of hexanal, together with LeHPL, a 13-HPL [67,68,115]. Another gene, *ADH2*, has been reported as positively related to the production of hexanol and (*Z*)-3-hexenol [116] while ADH1 showed in vitro activity in the conversion of hexanal into hexanol [117]. Figure 2 summarizes the complete biosynthetic pathway of the lipid-derived VOCs, according to the available literature.

### 3.2. QTLs for FA VOCs

Data collection identified a total number of 108 QTLs reported in 8 different studies and correlated with lipid volatiles biosynthesis (Table S1). Comparing these regions (see “Data Acquisition and Classification”), we identified 24 distinct QGRs (Figure 1, Table S1).

### 3.3. FA VOCs’ Candidate Genes

The collected data allowed the identification of 112 genes potentially involved in lipid VOCs biosynthesis (Table S1). Among them, 28 genes have been reported to be expressed in at least one fruit tissue (Table 3; [103]), or have been functionally characterized for their role in fatty acids metabolism. Eight lipoxygenases (LOXs) were identified as candidate genes expressed in tomato fruit. Among the identified LOXs, three have been reported for their association with lipid VOCs: *LoxC* (Solyc01g006540) has a major role in the biosynthesis of the most quantitatively prominent C5 and C6 lipid-derived VOCs, *LoxF* (Solyc01g006560) is involved in the production of fatty acid VOCs derived from 13-hydroperoxides [3,121].

Eleven alcohol dehydrogenases (ADH) were identified. ADHs are a family of enzymes associated with the interconversion of the aldehyde and alcohol forms of lipid volatiles in tomato and they have been reported to accumulate in the fruit during ripening [6,116]. Among them, Solyc06g059740 has been characterized in tomato fruit as ADH2 [116,122,123]. Solyc11g071290 was identified in the LIP21 QGR, a QTL for the earthy/mushroom odor type volatile 1-octen-3-one—which, unlike the major C6 VOCs, has rarely been suggested as an important fresh tomato fruit odorant, has a much lower concentration than C6 VOCs, but has an extremely low odor threshold of 0.005 nLּL^−1^ [124]. A structural variation in the promoter of this gene was reported in [26]. The wild allele was present in *S. pimpinellifolium* and *S. cerasiforme*, but was not found in heirloom tomatoes, suggesting selection against the wild allele during domestication. Although this gene showed a significant expression level in one fruit sample only (*S. pimpinellifolium* fruit at 4 DPA), we cannot exclude that allelic differences in this gene may be responsible for the 1-octen-3-one QTL identified by GWAS [99].

## 4. Branched-Chain Amino Acids Derivatives (BCAA VOCs)

Branched-chain amino acid (BCAA) derived compounds are highly volatile compounds with a low molecular weight, some of which are considered important in the development of tomato aroma, e.g., 3- and 2-methylbutanal (aldehydic/chocolate/musty odor types), 3- and 2-methylbutanol (roasted/fermented odor types), and 2-isobutyl-thiazole (green/wasabi odor), which is partly derived from BCAA [10,12,16].

### 4.1. Biosynthesis of BCAA VOCs

**Figure 3:**
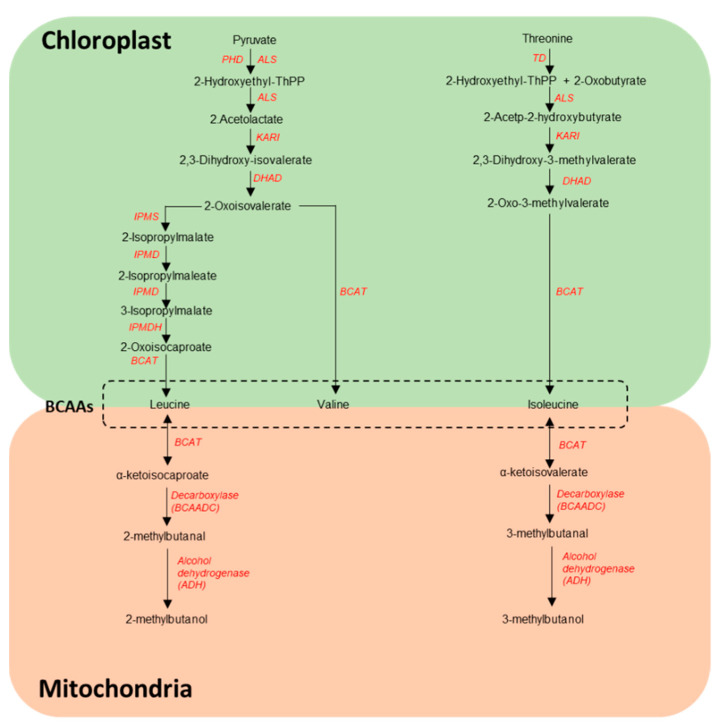
Overview of the proposed BCAA VOCs pathway, adapted from [94,130]. TD: threonine dehydratase; ALS: acetolactate synthase; KARI: ketol-acid reductoisomerase; DHAD: dihydroxy-acid dehydratase; BCAT: branched-chain aminotransferase; IPMS: 2-isopropylmalate synthase; IPMD: 3-isopropylmalate dehydratase; IPMDH: 3-isopropylmalate dehydrogenase; PDH: pyruvate dehydrogenase.

Even though the relationship between BCAA VOCs and the tomato aroma is clear, the exact molecular mechanism underlying their quantitative variation in tomato fruit is not fully understood. In general, BCAA VOCs originate from the branched-chain amino acid (BCAA) pathway in many organisms including plants. BCAA biosynthesis has been studied well in plants and it takes place in the chloroplast where leucine and valine are synthesized from pyruvate and isoleucine from threonine via several enzymatic reactions (Figure 3). Catabolism of BCAAs, which occurs in mitochondria is believed to be the source of BCAA VOCs in tomato fruit. It has been suggested that the first step in the catabolic pathway leading to BCAA VOCs is the reversible conversion of branched-chain amino acids (leucine, isoleucine) into their corresponding α-ketoacids by means of branched-chain amino acid aminotransferases (BCATs; [125]). The importance of BCATs in the degradation of BCAAs has been demonstrated in *Arabidopsis* [126]. In tomato different members of the BCAT family have been shown to mediate either synthetic or catabolic reactions of BCAAs [94,127,128,129]. The products of the reversible BCAT-mediated BCAA deamination—α-ketoacids—have been suggested to be the likely precursors for BCAA VOCs [128], which then could be produced through the combined action of various classes of candidate enzymes (Figure 3), such as α-ketoacid dehydrogenases, decarboxylases and alcohol/aldehyde dehydrogenases [130].

### 4.2. QTLs for BCAA VOCs

Data collection identified a total number of 129 QTLs reported by seven different authors and correlated with BCAA volatiles biosynthesis (Figure 1; Table S2). These QTLs were classified into 26 distinct QGRs (Table S2).

### 4.3. BCAA VOC Candidate Genes

Table S2 reveals 75 genes that were identified as potentially involved in the BCAA VOCs metabolism, 28 of which have been reported to be expressed in at least one fruit tissue [103]. An overview of the 30 selected candidate genes is shown in Table 4, including genes expressed in fruits plus two functionally characterized genes (*BCAT2* and *BCAT4*; see below).

Among the genes identified as expressed in tomato fruit, ten belong to enzymatic families that have been associated with BCAA biosynthesis: four genes were annotated as pyruvate dehydrogenases (PDH), three as 3-isopropylmalate dehydratases (IPMD), two as ketol-acid reductoisomerase (KARI) and one as 2-isopropylmalate synthase (IPMS). Reverse genetics analysis of Solyc06g060790 (*IPMD*) revealed that this gene influences the BCAA content in tomato fruit, while a similar analysis failed to support such a conclusion in case of Solyc07g053280 (*KARI*) [94]. Branched chain amino acid aminotransferases (BCATs) can be involved in the last step of BCAA anabolism and/or in the first step of BCAA catabolism depending on their subcellular localization (chloroplast or mitochondrion, respectively). Six BCAT genes were reported as candidate genes in Table 4, of which five (*BCAT1*, *2*, *3*, *4* and *7*) have been functionally characterized [112,114]. Although *BCAT2*, *4* and *7* were located outside the QTL intervals, they were included in this review for completeness. *BCAT1* and *BCAT2* were shown to be located in mitochondria and involved in the catabolism of BCAAs, while *BCAT3* and *BCAT4* were shown to be located in chloroplasts and involved in BCAA biosynthesis. In turning and ripe fruits of cv. M82 *BCAT1* expression was up to 10-fold higher compared to *BCAT2*, *3* and *4*. Although *BCAT2*, *3* and *4* showed low, but detectable expression in fruits, their expression was much higher in leaves (*BCAT2* and 3) or inflorescence (*BCAT4*) in this tomato background [127]. According to the RNAseq data present in the TomExpress database [103], these three genes are hardly (*BCAT3*) or not at all expressed in tomato fruits (*BCAT2* and *4*). Although its subcellular location is unclear, *BCAT7* was proposed to play a role in BCAA degradation [128]. Finally, we identified eleven alcohol dehydrogenases (ADH) expressed in tomato fruit in the BCAA QGRs. These may play a role in BCAA catabolism, although their functional characteristics remain to be demonstrated.

The available tomato pangenome [26] was investigated for the presence of non-reference (non cv. Heinz) promoter regions for the abovementioned genes. Interestingly, a non-reference allele was reported for Solyc04g063350, annotated as 3-methyl-2-oxobutanoate dehydrogenase, an enzymatic class that has been described as involved in the BCAA catabolism, which involves the decarboxylation of branch chain amino acids [94,129,131]. This gene has recently been named *FLORAL4* after both genetic and functional studies revealed that its involvement was not restricted to BCAA catabolism, but this gene also controlled the quantitative variation of floral phenolic-derived VOCs derived from catabolism of the aromatic amino acid phenylalanine (see below; [102]). Among the investigated tomato varieties, the cultivars harboring the ”wild” allele showed significantly lower gene expression than the ones presenting the domesticated promoter, suggesting a positive selection for *FLORAL4* expression during tomato domestication [26].

## 5. Carotenoid-Derived VOCs

Carotenoid-derived volatiles (apocarotenoid VOCs) are perceived as “floral” and accumulate in low amounts in tomato fruit [11]. However, due to their low odor thresholds [10], they have been reported to positively correlate with tomato flavour acceptability [15]. There are two groups of carotenoid VOCs that are relevant for tomato aroma [21,132]: (i) cyclic carotenoid volatiles, such as β-ionone and β-damascenone (both floral odor type); and (ii) open-chain carotenoid-derived volatiles, such as 6-methyl-5-hepten-2-one, geranial (both citrus odor type) and geranylacetone (floral odor type).

### 5.1. Biosynthesis

**Figure 4:**
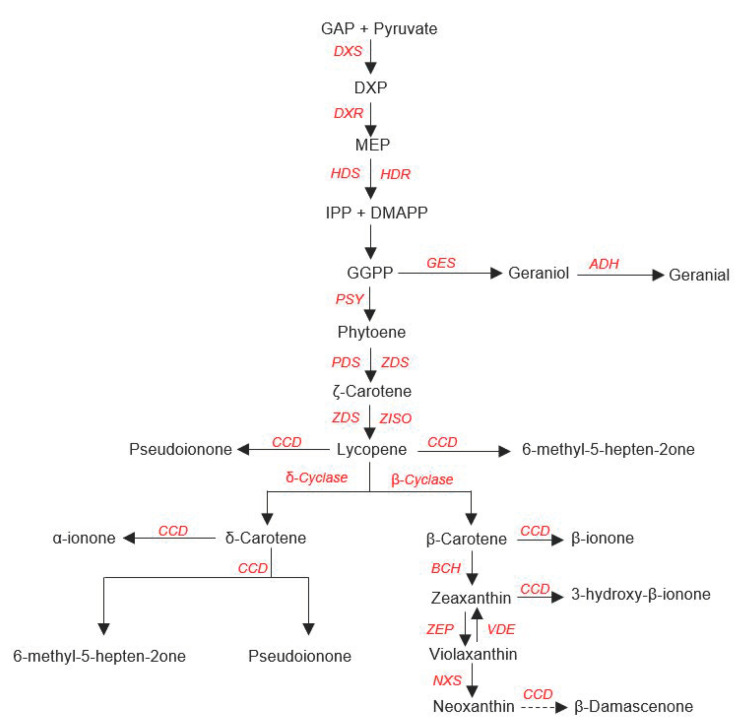
Overview of the carotenoid VOCs pathway, adapted from [135,141,142].

Apocarotenoid volatiles are produced in plastids [104] through the cleavage of carotenoids by carotenoid cleavage dioxygenases, like LeCCD1A and LeCCD1B [133], which are particularly expressed during fruit ripening [132]. This family of genes has been reported in other species to act both on cyclic carotenoids (at the 9′,10′ position) and open-chain carotenoids—at the (5′,6′), (7′,8′), or (9′,10′) positions-, leading to the production of the carotenoid VOCs (Figure 4; [133,134,135,136,137,138,139]). Although many structural genes in the carotenoid biosynthetic and cleavage pathway are known, the regulation of the carotenoid pathway is still unclear. In this respect it has been proposed that the loss of membrane integrity during the ripening-dependent conversion of chloroplasts into chromoplasts may be a key mechanism in their regulation, as this process may lead to the release of the carotenoids in the cytoplasm, were they will react with cytoplasmatic cleavage enzymes [15,140]. The available knowledge of the carotenoid biosynthetic and cleavage pathway and the underlying biosynthetic genes (Figure 4) is very helpful for the identification of candidate genes within QTLs for carotenoid-derived VOCs in tomato fruit.

### 5.2. QTLs for Apocarotenoid VOCs

Data collection identified a total number of 42 QTLs reported in 8 different articles and correlated with apocarotenoid volatiles biosynthesis (Figure 1; Table S3). Comparing these regions (see “Data Acquisition” paragraph for methodology), we identified 19 distinct QGRs (Table S3).

### 5.3. Apocarotenoid VOCs’ Candidate Genes

Twenty-three genes were identified as potentially associated with apocarotenoid VOCs metabolism (Table S3). For nineteen genes there is functional evidence for their involvement in the carotenoid pathway and five of those (Solyc03g007960, Solyc03g031860, Solyc03g123760, Solyc06g036260, Solyc10g081650) have also been shown to be expressed in at least one fruit tissue based on [103]. Solyc06g036260 (Crtz1), Solyc03g114340 (DXR), Solyc03g123760 (PDS), Solyc04g040190 (LCY-B1) are located outside the QTL regions, but were included for completeness (Table 5; [137,143,144,145,146,147,148,149,150,151,152,153,154]).

Two genes have been annotated as 1-deoxy-D-xylulose 5-phosphate synthases (DXS), reported as first and key regulatory step of the MEP pathway required for the production of carotenoids [141,143,155]. Most of the structural genes of the carotenoid pathway are found within the QTL regions, including three phytoene synthases required for the production of phytoene, the first carotenoid of the pathway [144,149,156,157,158,159,160], followed by phytoene desaturase [152] and one carotenoid isomerase [161], required for the production of lycopene, two lycopene cyclases, involved in the production of α- and β-carotene [153,154,162,163], one carotene hydroxylase to form zeaxanthin [159,164], and finally one zeaxanthin epoxidase, an enzymatic class that converts zeaxanthin, precursor of 3-hydroxy-β-ionone, into violaxanthin [132,135,151,165]. As mentioned above, lycopene β-cyclase 1 (Solyc04g040190) was not associated with any of the apocarotenoid QGRs. In total four carotenoid cleavage dioxygenases (CCDs) were present in the QGRs. Two CCD’s were annotated as carotenoid cleavage dioxygenase 1A (CCD1A—Solyc01g087250) and 1b (CCD1B—Solyc01g087260) and have been shown to be directly involved in carotenoid VOCs accumulation in tomato [132,138], one was annotated as carotenoid cleavage dioxygenase 2 (CCD2—Solyc01g087270), reported to be involved in the formation of carotenoid VOCs in Crocus sativus [137], and one as carotenoid cleavage dioxygenase 7 (CCD7—Solyc01g090660), associated with β-carotene cleavage to carlactone [166]. Two CDDs (CCD4A—Solyc08g075480, CCD4B—Solyc08g075490) have been reported to be associated with tomato fruit color, flavour, and aroma [167,168,169], but were not comprised in any identified QGR.

Phytoene synthase 2 (PSY2—Solyc02g081330) represents an exception to our candidate gene mining approach. This gene has been annotated at 45Mb on chr 2 and is located between QGRs APO3-4. Although this gene is not comprised among the regions identified by this review, its activity has been associated with carotenoid production in fruit in other Solanaceae [170]. For this reason, it has been proposed here as potential candidate gene for tomato apocarotenoid VOCs.

A search for reported wild promoter regions [26] was performed for the above-mentioned candidate genes, identifying Solyc03g114340 (*DXR*—1-deoxy-D-xylulose-5-phosphate reductoisomerase) as the only candidate gene showing a wild allele for its promoter region in the available tomato pangenome. This non-reference allele consists of a 645 bp promoter located at 582 bp upstream of *DXR*, and has been associated with an occurrence frequency of 0.58 in the *Solanum pimpinellifolium* L. accessions investigated by [26]. On the other hand, its presence in the *Solanum lycopersicum* var. *cerasiforme* and *S. lycopersicum* heirlooms has been reported to be rare, with an occurrence frequency of 0.05 and 0.02 respectively. The cultivars presenting the wild allele showed a significantly higher expression of Solyc03g11434 compared to the ones harboring the common allele, suggesting a selection against a higher expression of *DXR* during tomato domestication.

## 6. Phenylalanine-Derived Volatiles (Phe VOCs)

Phenolic and phenylpropanoid volatiles originate from the catabolism of phenylalanine. Phenolic VOCs include compounds such as phenylacetaldehyde, 2-phenylethanol, 1-nitro-2-phenylethane and 2-phenylacetonitrile (benzylnitrile; all floral odor type) which have been reported to affect consumer liking of tomato fruit, [8,12,16,17]. However, the effect of these phenolic compounds on flavour and consumer preference seems not easy to predict, since some studies show positive while others show negative effects of these compounds on consumer liking [13,92,172]. This apparent inconsistency may be caused by differences in the concentrations of these compounds in the tomato materials studied. The main phenylpropanoid VOCs in tomato are guaiacol, methylsalicylate and eugenol. They are associated with a smoky, pharmaceutical aroma and are generally considered as off-flavours.

### 6.1. Biosynthesis of Phe VOCs

Phenolic volatiles (C_6_–C_2_) have been reported to have a high impact on tomato aroma [11]. Their biochemical pathway starts with decarboxylation of phenylalanine, leading to the production of phenylethylamine [93]. According to the proposed phenolic volatile biosynthetic pathway in tomato, phenylethylamine is the precursor for the synthesis of the two nitrogen-containing volatiles nitrophenylethane and benzylnitrile, as well as the production of phenylacetaldehyde. Extremely high phenylacetaldehyde levels were found in tomato introgression lines carrying the malodorous locus from S. pennellii on chromosome 8 [92]. The high phenylacetaldehyde production in this line was associated with the expression of AADC1A, AADC1B, and AADC2 located in the malodorous QTL region [93,173,174]. Transgenic approaches revealed that this family of genes was capable of decarboxylating phenylalanine, leading to phenylethylamine, the direct precursor of phenylacetaldehyde. The subsequent deamination of phenethylamine to produce phenylacetaldehyde has been reported to be related to an amine oxidase. Finally, 2-phenyethanol is produced from phenylacetaldehyde by means of two reductases, PAR1 and PAR2 [175,176,177]. These enzymes are not only reducing phenylacetaldehyde, but they are also able to catalyze the reduction of benzaldehyde and cinnamaldehyde to their respective alcohols as well [175]. More recently, FLORAL4 (Solyc04g063350—3-methyl-2-oxobutanoate dehydrogenase- has been fine mapped in a diversity panel of cultivated contemporary tomato varieties and tomato RIL populations, and associated with the floral phenolic volatiles accumulation in tomato fruit [102]. Based on the protein sequence FLORAL4 belongs to the mitochondrial 2-oxoisovalerate dehydrogenase/decarboxylase enzyme family which is involved in the catabolism of BCAAs and constitutes the E1 subunit of the BCKDC complex, catalyzing the decarboxylation of the BCAA deamination products in plants [131]. A complete knock-out of the FLORAL4 gene by CRISPR-Cas9-mediated gene editing in tomato plants led to a major depletion of the phenylalanine-derived volatiles as well as a notable depletion of BCAA VOCs. This suggests involvement of FLORAL4 in both BCAA and PHE VOC metabolism, possibly via decarboxylation of the corresponding amino or keto acids.

**Figure 5:**
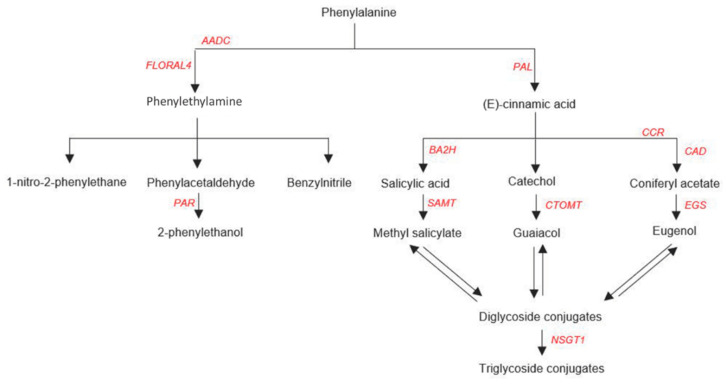
Overview of the phenolic and phenylpropanoid VOCs pathways, adapted from [11].

As mentioned above, phenylpropanoid volatiles (C_6_–C_3_) are the second group of phenylalanine derived VOCs. Although their biosynthesis in tomato has not been fully characterized, results from other species suggest that phenylpropanoid volatiles are derived from intermediates of the lignin pathway [178,179,180,181]. For example, eugenol has been reported in other species to be produced from coniferyl acetate by means of a eugenol synthase [182]. The mechanisms of methyl salicylate and guaiacol biosynthesis have been investigated in tomato [11]. Methyl salicylate can be produced from salicylic acid by means of SlSAMT1, an O-methyltransferase [183], while guaiacol may be produced from catechol, by means of the catechol-O-methyltransferase CTOMT1 [184]. Although CTOMT1 could not be connected to any of the QTL regions found, it has been included for completeness. Furthermore, the conjugation of these three compounds after their biosynthesis has been reported to be linked to the activity of two classes of enzymes: glycosyltransferases and glycosyl hydrolases. Among these classes of enzymes, NSGT1 was shown to prevent the wound-induced release of the smoky aroma associated with phenylpropanoid VOCs in ripening tomato fruit [185]. Figure 5 summarizes the metabolic reactions described above.

### 6.2. QTLs for Phe VOCs

Data collection identified a total number of 81 QTLs reported by eight different authors and correlated with phenylalanine-derived volatiles biosynthesis (Figure 2; Table S4). Comparing these regions (see “Data Acquisition” paragraph for methodology), we identified 24 distinct QGRs (Table S3).

### 6.3. Phe VOCs’ Candidate Genes

Seventy-five genes were identified as potentially associated with phenylalanine derived VOCs, including all candidate genes present in the QGRs plus the above-mentioned CTOMT1 (Solyc10g005060—Table S4). Among them, twenty-one have been reported to be expressed in at least one fruit tissue or were characterized for their role in VOCs metabolism (Table 6 and Table S4; [103]). In addition, the above-mentioned NSGT1, present in QGR PHEN17, was also included in Table 6. NSGT1 expression in tomato fruit was reported by [185], but this gene has not been predicted by the tomato genome and its genomic organization has only recently been resolved [65], and hence gene expression data for this gene could not be retrieved from the TomExpress database.

Among the genes identified as expressed in tomato fruit, one was annotated as 3-methyl-2-oxobutanoate dehydrogenase (Solyc04g063350), the above-mentioned FLORAL4 gene and was demonstrated to be the causal gene for the variation in phenolic VOCs in the PHEN7 QGR on chromosome 4.

One gene (SlFMO1—Solyc12g013690) was annotated as flavin-dependent monooxygenases, a class of enzymes that was shown to catalyze the hydroxylation of aromatic compounds in prokaryotes and plants [174,175,176]. This gene was suggested to play a role in the synthesis of nitrogenous phenolic volatiles, like 2-phenylacetonitrile and 1-nitro-2-phenylethane in tomato [186], although this needs experimental confirmation. Furthermore, we identified one primary amine oxidase (Solyc08g079430) that may potentially be involved in the production of 2-phenylacetaldehyde from phenethylamine [93].

We identified five phenylalanine ammonia-lyase (Solyc09g007890, Solyc09g007900, Solyc09g007910, Solyc09g007920, and Solyc10g086180) genes expressed in tomato fruit. These enzymes have been reported to be responsible for the first key step in phenylpropanoid metabolism, catalyzing the conversion of l-phenylalanine into trans-cinnamate [187].

Finally, six decarboxylase genes were reported to be expressed in tomato fruit during ripening [103,188]. Among them, Solyc08g066250 has been reported as SlHDC11 and has been associated with fruit-ripening in tomato [189]. Moreover, LeAADC1A (Solyc08g068680), together with LeAADC1B (Solyc08068610) and LeAADC2 (Solyc08g006740), have been related with the conversion of phenylalanine to phenylethylamine in a S. lycopersicum var. M82 × S. pennellii tomato IL population [93].

The tomato pangenome [26] was investigated for potential non-reference alleles in the promoter region of the identified candidate genes. A presence-absence variant was detected in the promoter of FLORAL4 (Solyc04g063350). This variant has an occurrence frequency of 0.74 in cultivated heirloom tomato and 0.64 in *S. lycopersicum* var. cerasiforme, while it occurs at a frequency of 0.02 only in *S. pimpinellifolium* WL. Gene expression analyses pinpointed a significant difference (*p*-value: 5.48 × 10^−6^) between accessions harboring the domesticated versus the wild allele, suggesting that the domesticated allele of FLORAL4 has a lower expression compared to the wild allele.

## 7. Concluding Remarks

In the last decades, a vast array of scientific studies has investigated the genetic components of tomato aroma in modern tomato cultivars and their relatives. However, the methodological differences between different studies, such as the source materials, the type of mapping population, the number of markers, the reference map used, the influence of environmental factors on trait performance and analytical variation in determination of the aroma phenotype, make it difficult for breeders and researchers to efficiently use the available data in their research. This review summarized the state of the art on the understanding of tomato aroma genetics and provides a tool that can be used by breeders and researchers to collect additional evidence for the robustness of their QTL data. The identified QGRs, especially when defined by the overlapping of QTLs from different sources, represent the regions that most likely contain genetic elements that regulate tomato aroma. The QGRs differ from each other by two major parameters—the size and the number of studies contributing to it. The smaller the size of a resulting QGR and the higher the number of studies referring to it suggests a high robustness of such a QGR in a breeding program. Some of the QGRs, however, appeared to be quite large. This most likely indicates the presence of multiple genetic factors controlling the levels of (related) aromatic compounds in such a region, although we cannot exclude the possibility that, in some cases, a large QGR is caused by inaccurate prediction of a QTL due to experimental and/or methodological differences, as mentioned above. For a breeder such large QGRs could indicate a need for further inspection and dissection of the respective QGR in selected donor material, using high-resolution (fine) mapping approaches.

There are various potential applications of these data in practical breeding and breeding research:

(i) The QTL information presented in this review can be directly used to support marker-assisted breeding programs aimed at introgressing large-effect aroma QTLs into elite germplasm, for example using the donors indicated in Table S5.
(ii) The current development of pangenome projects paves the way for a new step in tomato breeding research. Advances in computational genomics and long-read sequencing allow an easier and more comprehensive investigation of the genetic variation in tomato collections worldwide. This makes it possible to identify genetic elements that are missing in the reference genome and to discover and use novel markers—such as Structural Variants (SVs) and Present Absent Variants (PAVs) [190]. Thanks to the ongoing reduction in sequencing costs, large sets of genotypes can nowadays be re-sequenced, allowing the application of SV markers in GWAS projects [191,192]. Furthermore, SV identification in large sets of genotypes can lead to downstream breeding approaches. For example, SV-based linkage mapping can be applied by genotyping mapping populations using SV markers that showed polymorphism in the parental lines [193]. Furthermore, SV studies may help to get more insight in the mechanisms leading to a certain phenotype [26,65,194,195], pinpointing the best donors for a certain allele cross. By providing a comprehensive set of candidate genes for tomato aroma, our review may guide researchers and breeders in the selection of the most interesting genes that can be investigated for structural variation.
(iii) The data in this review can be used to support the identification and use of the key genes underlying these QTLs. In combination with available (pan)genomic and transcriptomic information candidate genes present in the QTL regions can be selected and tested for their effects in vivo using either stable transgenic approaches such as CRISPR-CAS9 mediated gene editing or quicker transient overexpression or silencing in tomato fruit. This may not only lead to the identification of the causal genes controlling a trait, but also to the detection of the causal genetic variants underlying trait variation. Such variants, also called functional markers, are the best possible molecular markers for MAS, since they are functionally linked to the trait rather than genetically linked and their use as marker does not need validation in other populations, which is always required with genetically-linked markers [196].
(iv) The information on large effect aroma QTLs provided in this review can alo be used to improve the performance of genomic prediction models, since both genetically-linked markers and in particular functional markers have been shown to significantly improve the prediction power of GP models compared to the use of random neutral markers [197].

Last but not least we hope that this review may facilitate the development of more tasty tomatoes.

## Figures and Tables

**Table 1:** Overview of the 21 main components of tomato aroma (adapted from [11]). * Odor descriptors were retrieved from the Good Scents Company database [18].

Class	Compound	Odor Descriptors *
Apocarotenoid	6-Methyl-5-hepten-2-one	Citrus, green, musty, lemongrass, apple
Apocarotenoid	Geranial	Sharp, lemon, sweet
Apocarotenoid	β-Damascenone	Apple, rose, honey, tobacco, sweet
Apocarotenoid	Geranylacetone	Fresh, green, fruity, waxy, rose, woody, magnolia, tropical
Apocarotenoid	β-Ionone	Floral, woody, sweet, fruity, berry, tropical, beeswax
BCAA	3-Methylbutanal	Ethereal, aldehydic, chocolate, peach, fatty
BCAA	2-Methylbutanal	Musty, cocoa, coffee, nutty
BCAA	3-Methylbutanol	Fusel, oil, alcoholic, whiskey, fruity, banana
BCAA	2-Isobuthylthiazole	Green, wasabi, privet, tomato, leaf, earthy, vegetable, metallic
Lipid	1-Penten-3-one	Pungent, peppery, mustard, garlic, onion
Lipid	(*Z*)-3-Hexenal	Green, fatty, grassy, weedy, fruity, apple
Lipid	Hexenal	Sweet, almond, fruity, green, leafy, apple, plum, vegetable
Lipid	(*E*)-2-Hexenal	Sharp, fresh, leafy, green, clean, fruity, spicy, herbal
Lipid	(*E*)-2-Heptenal	Pungent, green, vegetable, fresh, fatty
Lipid	(*E*,*E*)-2.4-Decadienal	Oily, cucumber, melon, citrus, pumpkin, nut, meat
Phenolic	Phenylacetaldehyde	Green, sweet, floral, hyacinth, clover, honey, cocoa
Phenolic	2-Phenylethanol	Sweet, floral, fresh, bready, rose, honey
Phenolic	1-Nitro-2-phenylethane	Flower, spice
Phenylpropanoid	Guaiacol	Phenolic, smoke, spice, vanilla, woody
Phenylpropanoid	Methyl salicylate	Wintergreen, mint
Phenylpropanoid	Eugenol	Sweet, spicy, clove, woody

**Table 2:** List of the articles reporting QTLs for tomato aroma included in the review. RILs: Recombinant Inbred Lines; BCs: Backcross population; ILs: Introgression Lines; GWAS: Genome-Wide Association Studies; DP: Diversity Panel.

Author, Year	Population	References
Saliba-Colombani et al., 2001	RILs	[90]
Fulton et al., 2002	BCs	[91]
Tadmor et al., 2002	ILs	[92]
Tieman et al., 2006	ILs	[93]
Mathieu et al., 2009	ILs	[6]
Kochevenko & Fernie, 2011	ILs	[94]
Ruggieri et al., 2014	GWAS	[95]
Sauvage et al., 2014	GWAS	[74]
Capel et al., 2015	RILs	[96]
Zhao et al., 2016	GWAS	[97]
Baldina et al., 2016	GWAS	[98]
Bauchet et al., 2017	GWAS	[99]
Tieman et al., 2017	GWAS	[25]
Garbowicz et al., 2018	ILs	[100]
Kimbara et al., 2018	RILs	[101]
Tikunov et al., 2020	F2, F6, DP	[102]

**Table 3:** List of the genes identified as potentially responsible for the lipid VOCs QTLs reported in literature. Genes that have been functionally characterized are highlighted in bold in the table. The last column reports whether the gene has been reported to be expressed in fruit (Y/N—[103]), for a complete overview of the gene expression see Table S1.

Solyc ID	Name	QGR	Start (bp)	Annotation	Reference	Expressed (Y/N)
**Solyc01g006540**	***loxC***	**LIP1**	**1,113,718**	**Lipoxygenase**	[68]	**Y**
**Solyc01g006560**	***loxF***	**LIP1**	**1,128,815**	**Lipoxygenase**	[121]	**N**
Solyc01g099210	-	**LIP1**	89,509,072	Lipoxygenase		Y
Solyc01g108150	-	LIP2	95,505,678	Oxidoreductase zinc-binding dehydrogenase family protein		Y
Solyc02g090930	-	LIP2	52,398,542	Lipase		Y
Solyc03g093360	-	LIP3	54,632,041	Lipoxygenase		Y
Solyc03g095360	-	LIP5	56,412,064	Alcohol dehydrogenase zinc-binding		Y
Solyc03g111550	-	LIP5	62,149,409	GDSL esterase/lipase		Y
Solyc04g010250	-	LIP6	3,577,656	Lipase-like protein		Y
Solyc04g054980	-	LIP7	53,517,056	Lipoxygenase homology domain-containing protein 1		Y
Solyc04g054990	-	LIP7	53,522,447	Lipoxygenase homology domain-containing protein 1		Y
Solyc04g064710	-	LIP7	55,858,388	Alcohol dehydrogenase 2		Y
Solyc05g005480	*EO QR*	LIP9	352,211	Oxidoreductase zinc-binding dehydrogenase		Y
Solyc05g009390	-	LIP9	3,544,526	Lipase-like protein		Y
**Solyc06g059740**	***ADH2***	**LIP11**	**37,606,747**	**Alcohol dehydrogenase 2**		**Y**
Solyc07g045090	-	LIP13	58,206,449	Alcohol dehydrogenase zinc-binding domain protein	[116]	Y
Solyc08g014000	*LOXA*	LIP14	3,516,113	Lipoxygenase		Y
Solyc09g059030	-	LIP17	53,010,842	Alcohol dehydrogenase zinc-containing		Y
Solyc09g059040	-	LIP17	53,050,984	Alcohol dehydrogenase zinc-containing		Y
Solyc09g091050	-	LIP18	70,389,731	Lipase		Y
Solyc11g010960	-	LIP20	4,006,687	Alcohol dehydrogenase		Y
Solyc11g011330	-	LIP20	4,375,432	Cinnamyl alcohol dehydrogenase		Y
Solyc11g065530	-	LIP21	50,971,492	Lipase (Fragment)		Y
Solyc11g071290	-	LIP21	54,819,572	Alcohol dehydrogenase		Y
Solyc12g010950	-	LIP22	3,827,848	Alcohol dehydrogenase zinc-containing		Y
Solyc12g011040	-	LIP22	3,894,074	Lipoxygenase		Y
Solyc12g096760	-	LIP24	65,539,079	Alcohol dehydrogenase zinc-containing		Y
Solyc12g096780	-	LIP24	65,557,520	Mitochondrial trans-2-enoyl-CoA reductase		Y

**Table 4:** List of the genes identified as potentially responsible for the BCAA VOCs QTL reported in literature. Genes that have been functionally characterized are highlighted in bold in the table. The last column reports whether the gene has been reported to be expressed in fruit (Y/N—[103]); for a complete overview of the gene expression see Table S2.

Solyc ID	Name	QGR	Start (bp)	Annotation	Family	References	Expressed (Y/N)
**Solyc01g098700**	***SlBCAT7***		**89,114,957**	**Branched-chain-amino-acid aminotransferase 7**	**BCAT**	[128]	**Y**
**Solyc02g091970**	***SlBCAT3***	**BCAA4**	**53,178,240**	**Branched-chain-amino-acid aminotransferase 3**	**BCAT**	[127]	**Y**
Solyc03g005730		BCAA5	509,260	3-isopropylmalate dehydratase large subunit 2	IPMD		Y
Solyc03g007200		BCAA5	1,775,153	Oxidoreductase zinc-containing alcohol dehydrogenase family	ADH		Y
**Solyc03g043880**	***SlBCAT4***		**7,542,329**	**Branched-chain amino acid aminotransferase 4**	**BCAT**	[127]	**N**
Solyc03g097680		BCAA6	60,009,332	Pyruvate dehydrogenase E1 component subunit β	PDH		Y
Solyc04g008590		BCAA7	2,195,990	Pyruvate dehydrogenase E1 component subunit β	PDH		Y
**Solyc04g063350**	***FLORAL4***	**BCAA9**	**55,462,543**	**3-methyl-2-oxobutanoate dehydrogenase**		[102]	**Y**
Solyc04g064710		BCAA9	55,858,388	Alcohol dehydrogenase 2	ADH		Y
Solyc05g005480	*EO QR*	BCAA10	352,211	Oxidoreductase zinc-binding dehydrogenase	ADH		Y
Solyc06g059880		BCAA11	37,781,458	Acetolactate synthase	ALS		Y
**Solyc06g060790**		**BCAA11**	**38,814,018**	**3-isopropylmalate dehydratase small subunit**	**IPMD**	[94]	**N**
**Solyc07g021630**	***SlBCAT2***		**20,381,557**	**Branched-chain amino acid aminotransferase 2**	**BCAT**	[127]	**Y**
Solyc07g045090		BCAA13	58,206,449	Alcohol dehydrogenase zinc-binding domain protein	ADH		Y
Solyc07g053280		BCAA14	61,749,215	Ketol-acid reductoisomerase	KARI		Y
Solyc07g061940		BCAA14	64,814,098	Acetolactate synthase	ALS		Y
Solyc08g014130		BCAA15	3,972,785	2-isopropylmalate synthase 1	IPMS		Y
Solyc09g008670	*TD*	BCAA17	2,123,840	Threonine ammonia-lyase biosynthetic	TD		Y
Solyc11g010960		BCAA21	4,006,687	Alcohol dehydrogenase	ADH		Y
Solyc11g011330		BCAA21	4,375,432	Cinnamyl alcohol dehydrogenase	ADH		Y
Solyc11g071280	*LOC778238*	BCAA24	54,809,918	Branched-chain amino acid aminotransferase like protein	BCAT		Y
Solyc11g071290		BCAA24	54,819,572	Alcohol dehydrogenase	ADH		Y
Solyc12g005860		BCAA25	490,745	3-isopropylmalate dehydratase large subunit	IPMD		Y
Solyc12g009400		BCAA25	2,682,210	Pyruvate dehydrogenase E1 component α subunit	PDH		Y
Solyc12g009410		BCAA25	2,687,666	Pyruvate dehydrogenase E1 component α subunit	PDH		Y
Solyc12g010840		BCAA25	3,773,065	Ketol-acid reductoisomerase	KARI		Y
Solyc12g010950		BCAA25	3,827,848	Alcohol dehydrogenase zinc-containing	ADH		Y
Solyc12g088220	*SlBCAT1*	BCAA26	63,663,328	Branched-chain-amino-acid aminotransferase 1	BCAT	[127]	Y
Solyc12g096760		BCAA26	65,539,079	Alcohol dehydrogenase zinc-containing	ADH		Y
Solyc12g096780		BCAA26	65,557,520	Mitochondrial trans-2-enoyl-CoA reductase	ADH		Y

**Table 5:** List of the genes identified as potentially responsible for the carotenoid VOCs QTL reported in literature. Genes that have been functionally characterized are highlighted in bold in the table. The last column reports whether the gene has been reported to be expressed in fruit (Y/N—[103]); for a complete overview of the gene expression see Table S3.

Solyc ID	Name	QGR	Start (bp)	Annotation	References	Expressed (Y/N)
**Solyc01g005940**	***PSY3***	**APO1**	**613,955**	**Phytoene synthase 3**	[171]	**N**
**Solyc01g067890**	***DXS1***	**APO2**	**76,868,469**	**1-Deoxy-d-xylulose 5-phosphate synthase 1**	[145,155]	**N**
**Solyc01g087250**	***CCD1A***	**APO2**	**82,184,585**	**Carotenoid cleavage dioxygenase 1A**	[132,138]	**N**
**Solyc01g087260**	***CCD1B***	**APO2**	**82,196,996**	**Carotenoid cleavage dioxygenase 1B**	[132,138]	**N**
**Solyc01g087270**	***CCD2***	**APO2**	**82,209,237**	**Carotenoid cleavage dioxygenase 2**	[137]	**N**
**Solyc01g090660**	***CCD7***	**APO2**	**84,307,951**	**Carotenoid cleavage dioxygenase 7**	[166]	**N**
Solyc02g081330	*PSY2*	APO3/4	45,335,358	Phytoene synthase 2		N
**Solyc02g090890**	***ZEP***	**APO4**	**52,369,708**	**Zeaxanthin epoxidase, 2C chloroplastic**	[151]	**N**
**Solyc03g007960**	***CrtZ-2***	**APO5**	**2,447,872**	**β-Carotene hydroxylase 2**	[164]	**Y**
**Solyc03g031860**	***PSY1***	**APO5**	**4,326,134**	**Phytoene synthase 1**	[171]	**Y**
**Solyc03g114340**	***DXR***		**64,347,674**	**1-Deoxy-d-xylulose-5-phosphate reductoisomerase**	[143]	**N**
**Solyc03g123760**	***PDS***		**70,501,011**	**Phytoene desaturase**	[152]	**Y**
**Solyc04g040190**	***LCY-B1***		**11,947,053**	**Lycopene β-cyclase 1**	[153]	**N**
**Solyc06g036260**	***CrtZ-1***		**25,742,578**	**β-Carotene hydroxylase 1**	[162]	**Y**
**Solyc06g074240**	***LCY-B2***	**APO12**	**45,898,227**	**Lycopene β-cyclase 2**	[153]	**N**
**Solyc08g075480**	***CCD4A***		**59,627,153**	**Carotenoid cleavage dioxygenase 4A**	[167]	**N**
**Solyc08g075490**	***CCD4B***		**59,643,898**	**Carotenoid cleavage dioxygenase 4B**	[167]	**N**
**Solyc10g079480**	***LCY-b***	**APO17**	**61,024,821**	**Lycopene β-cyclase**	[153]	**N**
**Solyc10g081650**	***CRTISO***	**APO17**	**62,682,440**	**Carotenoid isomerase, 2C chloroplastic**	[161]	**Y**
**Solyc11g010850**	***DXS2***	**APO18**	**3,870,455**	**1-Deoxy-d-xylulose 5-phosphate synthase 2**	[145,155]	**N**

**Table 6:** List of the genes identified as potentially responsible for the phenylalanine-derived VOCs QTL reported in literature. Genes that have been functionally characterized are highlighted in bold in the table. The last column reports whether the gene has been reported to be expressed in fruit (Y/N—[103]); for a complete overview of the gene expression see Table S4.

Solyc ID	Name	QGR	Start (bp)	Annotation	References	Expressed (Y/N)
Solyc01g107910		PHEN3	95,296,421	Caffeoyl CoA 3-*O*-methyltransferase		Y
Solyc02g093270		PHEN5	54,178,272	Caffeoyl CoA *O*-methyltransferase		Y
Solyc03g097700		PHEN6	60,029,697	*O*-methyltransferase		Y
Solyc03g111830		PHEN6	62,444,512	*O*-methyltransferase		Y
**Solyc04g063350**	***FLORAL4***	**PHEN7**	**55,462,543**	**3-methyl-2-oxobutanoate dehydrogenase**	[102]	**Y**
Solyc04g071140		PHEN7	58,070,304	Decarboxylase family protein		Y
Solyc05g013440		PHEN8	6,501,962	Primary amine oxidase		Y
Solyc06g059840	*LOC778303*	PHEN9	37,729,682	3-methyl-2-oxobutanoate dehydrogenase		Y
**Solyc08g006740**	***AADC2***	**PHEN14**	**1,295,712**	**Decarboxylase family protein**	[175]	**N**
Solyc08g066240		PHEN15	54,722,572	Decarboxylase family protein		Y
**Solyc08g066250**	***Hdc***	**PHEN15**	**54,745,923**	**Decarboxylase family protein**		**Y**
Solyc08g068600		PHEN15	57,730,921	Decarboxylase family protein		Y
**Solyc08g068610**	***AADC1B***	**PHEN15**	**57,740,004**	**Decarboxylase family protein**	[175]	**Y**
**Solyc08g068680**	***AADC1A***	**PHEN15**	**57,812,621**	**Decarboxylase family protein**	[175]	**N**
Solyc08g079430		PHEN15	62,954,528	Primary amine oxidase		Y
Solyc09g007890		PHEN16	1,413,536	Phenylalanine ammonia-lyase		Y
Solyc09g007900		PHEN16	1,419,041	Phenylalanine ammonia-lyase		Y
Solyc09g007910		PHEN16	1,429,132	Phenylalanine ammonia-lyase		Y
Solyc09g007920		PHEN16	1,435,451	Phenylalanine ammonia-lyase		Y
**Solyc09g091550**	**SAMT1**	**PHEN17**	**70,802,564**	**Salicylate methyltransferase 1**	[183]	**N**
	NSGT1	PHEN17	64,653,692	Glycosyltransferase	[185]	Y
**Solyc10g005060**	***CTOMT1***		**53,330**	**Catechol-*O*-methyltransferase 1**	[184]	**Y**
Solyc10g085830		PHEN18	64,899,145	*O*-methyltransferase 1		Y
Solyc10g086180		PHEN18	65,098,335	Phenylalanine ammonia-lyase		Y
**Solyc12g013690**	***SlFMO1***	**PHEN22**	**4,532,510**	**Monooxygenase FAD-binding protein**	[175]	**Y**

## Data Availability

Data available in a publicly accessible repository.

## Supplementary Materials

The following are available online at https://www.mdpi.com/2073-4425/12/2/226/s1, Figures S1–S4: Gene expression (Euclidean heatmap) of the candidate genes identified in this review; Tables S1–S4: QGRs, Candidate Genes and Candidate Genes expressed in tomato fruit for the four classes of VOCs; Table S5: QTLs reported in literature (biosynthetic pathway, QGR, the QTL’s original name, related compound, chromosome, correlated markers, their position in cM and bp, their *p*-value, their LOD score, the SolycID of the gene in which the markers have been found, the percentage of explained variation by the QTL, the effect, donor parent, the crossing population or association panel used, and the reference of the primary resource).
