# Supplementary material for: The Genetic Basis of Tomato Aroma
Source: Genes (Basel). 2021 Feb 4;12(2):226. doi: 10.3390/genes12020226 (PMC7915847; doi:10.3390/genes12020226)
Supplement: Supplementary file 1 [file genes-12-00226-s001.zip › Supp.Figures/S1.2.pdf]

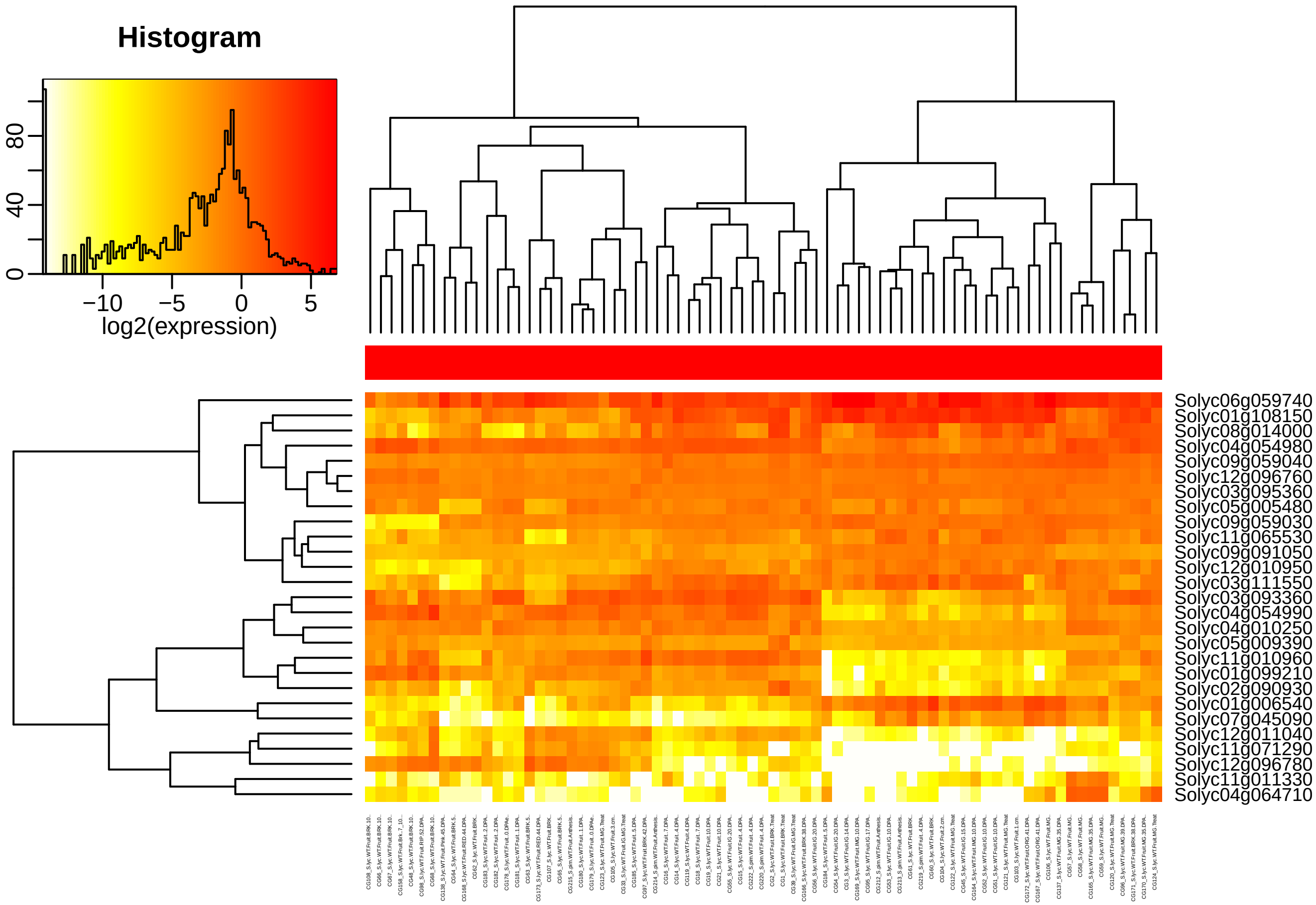
Histogram
0
40
80
−10
−5
0
5
log2(expression)
Solyc06g059740
Solyc01g108150
Solyc08g014000
Solyc04g054980
Solyc09g059040
Solyc12g096760
Solyc03g095360
Solyc05g005480
Solyc09g059030
Solyc11g065530
Solyc09g091050
Solyc12g010950
Solyc03g111550
Solyc03g093360
Solyc04g054990
Solyc04g010250
Solyc05g009390
Solyc11g010960
Solyc01g099210
Solyc02g090930
Solyc01g006540
Solyc07g045090
Solyc12g011040
Solyc11g071290
Solyc12g096780
Solyc11g011330
Solyc04g064710
