# Supplementary material for: The Genetic Basis of Tomato Aroma
Source: Genes (Basel). 2021 Feb 4;12(2):226. doi: 10.3390/genes12020226 (PMC7915847; doi:10.3390/genes12020226)
Supplement: Supplementary file 1 [file genes-12-00226-s001.zip › Supp.Figures/S2.2.pdf]

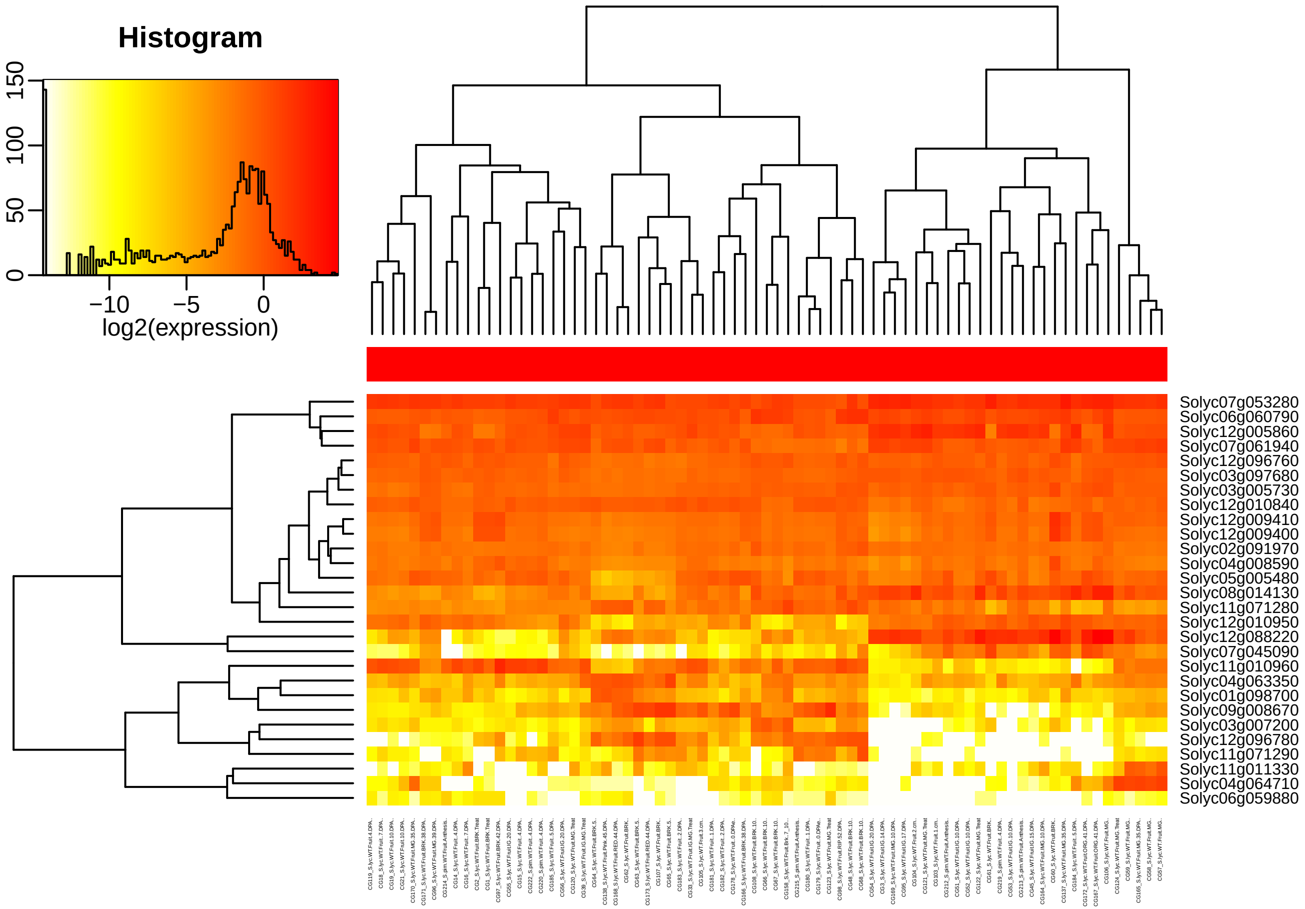
Histogram
0
50
100
150
−10
−5
0
log2(expression)
Solyc07g053280
Solyc06g060790
Solyc12g005860
Solyc07g061940
Solyc12g096760
Solyc03g097680
Solyc03g005730
Solyc12g010840
Solyc12g009410
Solyc12g009400
Solyc02g091970
Solyc04g008590
Solyc05g005480
Solyc08g014130
Solyc11g071280
Solyc12g010950
Solyc12g088220
Solyc07g045090
Solyc11g010960
Solyc04g063350
Solyc01g098700
Solyc09g008670
Solyc03g007200
Solyc12g096780
Solyc11g071290
Solyc11g011330
Solyc04g064710
Solyc06g059880
