# Supplementary material for: The Genetic Basis of Tomato Aroma
Source: Genes (Basel). 2021 Feb 4;12(2):226. doi: 10.3390/genes12020226 (PMC7915847; doi:10.3390/genes12020226)
Supplement: Supplementary file 1 [file genes-12-00226-s001.zip › Supp.Figures/S3.1.pdf]

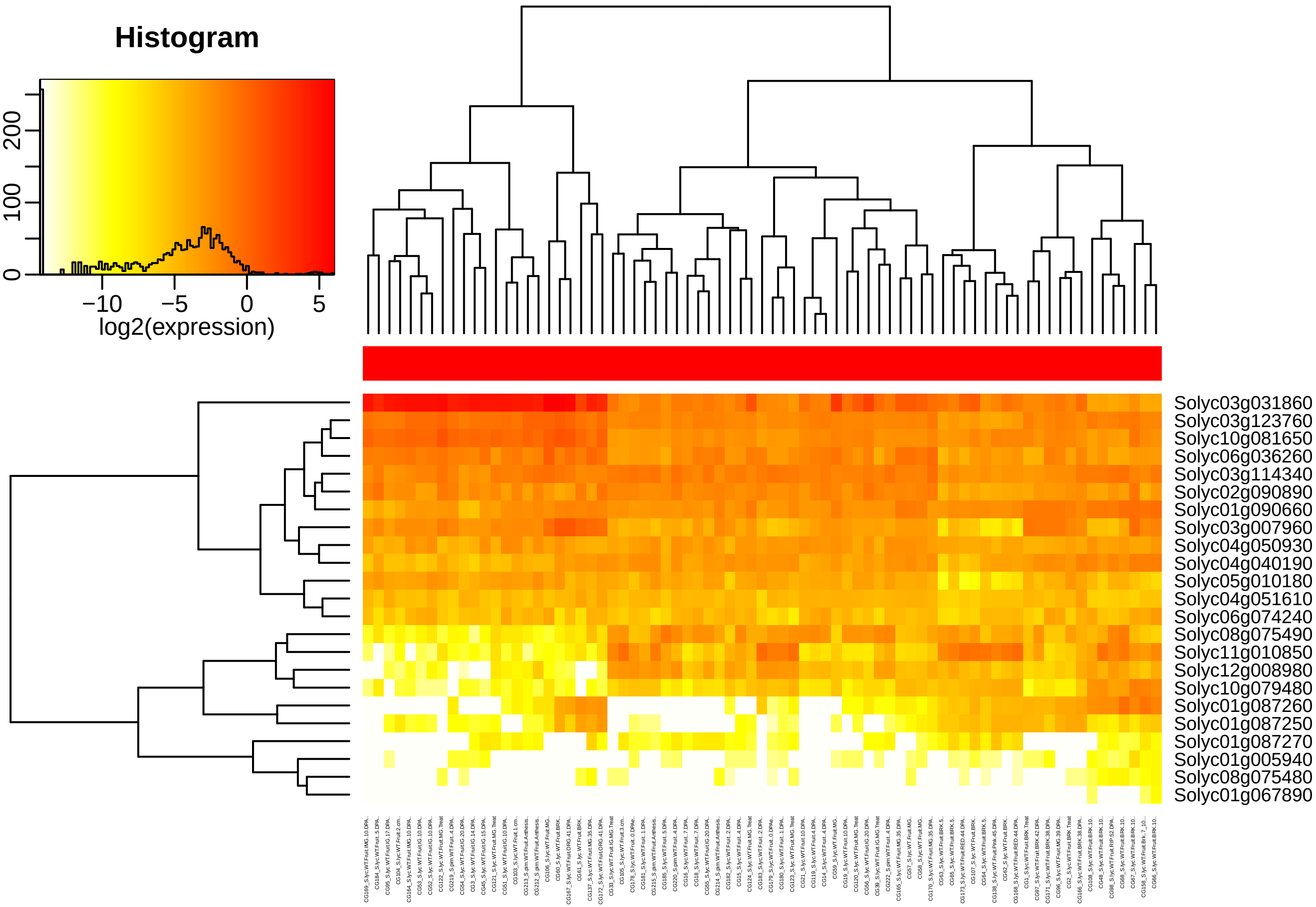
Histogram
0
100
200
−10
−5
0
5
log2(expression)
Solyc03g031860
Solyc03g123760
Solyc10g081650
Solyc06g036260
Solyc03g114340
Solyc02g090890
Solyc01g090660
Solyc03g007960
Solyc04g050930
Solyc04g040190
Solyc05g010180
Solyc04g051610
Solyc06g074240
Solyc08g075490
Solyc11g010850
Solyc12g008980
Solyc10g079480
Solyc01g087260
Solyc01g087250
Solyc01g087270
Solyc01g005940
Solyc08g075480
Solyc01g067890
