# Supplementary material for: The Genetic Basis of Tomato Aroma
Source: Genes (Basel). 2021 Feb 4;12(2):226. doi: 10.3390/genes12020226 (PMC7915847; doi:10.3390/genes12020226)
Supplement: Supplementary file 1 [file genes-12-00226-s001.zip › Supp.Figures/S4.2.pdf]

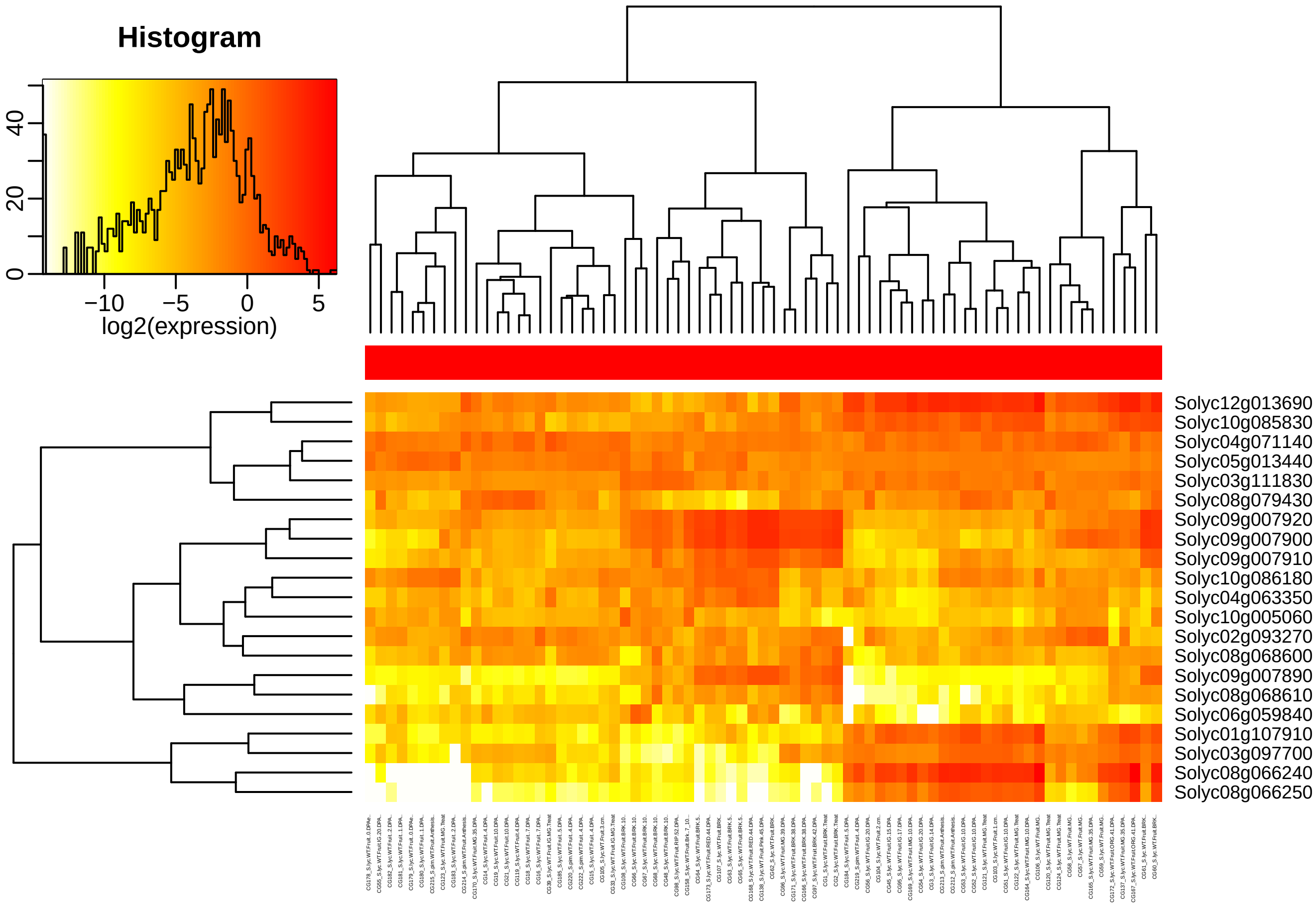

Histogram
0
20
40
−10
−5
0
5
log2(expression)
Solyc12g013690
Solyc10g085830
Solyc04g071140
Solyc05g013440
Solyc03g111830
Solyc08g079430
Solyc09g007920
Solyc09g007900
Solyc09g007910
Solyc10g086180
Solyc04g063350
Solyc10g005060
Solyc02g093270
Solyc08g068600
Solyc09g007890
Solyc08g068610
Solyc06g059840
Solyc01g107910
Solyc03g097700
Solyc08g066240
Solyc08g066250
