# Supplementary figures and images for: The Genetic Basis of Tomato Aroma
Source: Genes (Basel). 2021 Feb 4;12(2):226. doi: 10.3390/genes12020226 (PMC7915847; doi:10.3390/genes12020226)

## Histogram

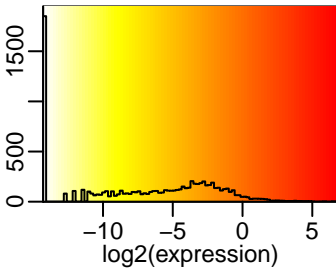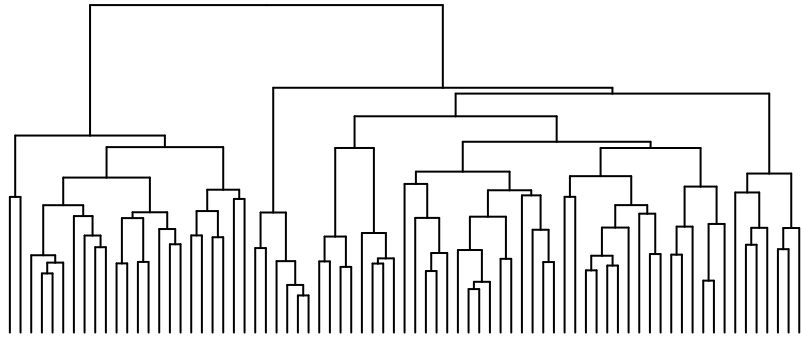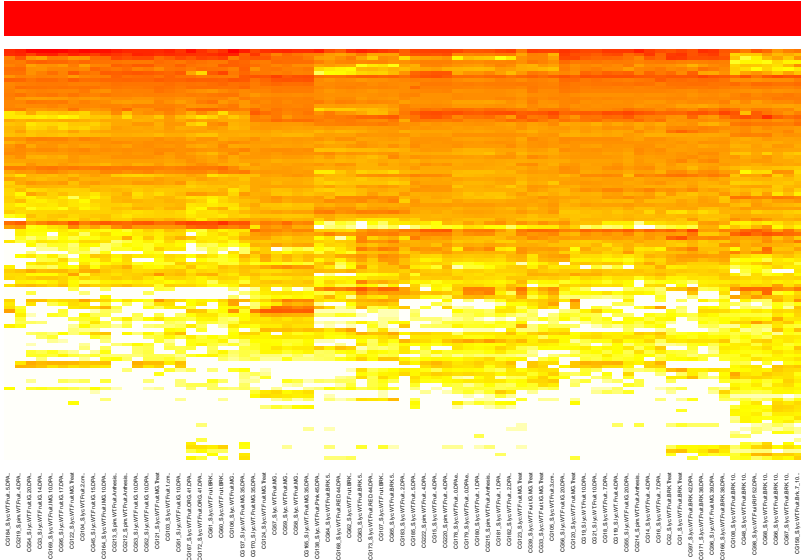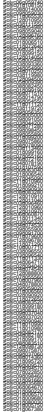

Supplement: Supplementary file 1 [file genes-12-00226-s001.zip › Supp.Figures/S1.1.pdf]

## Histogram

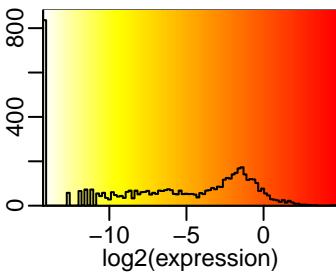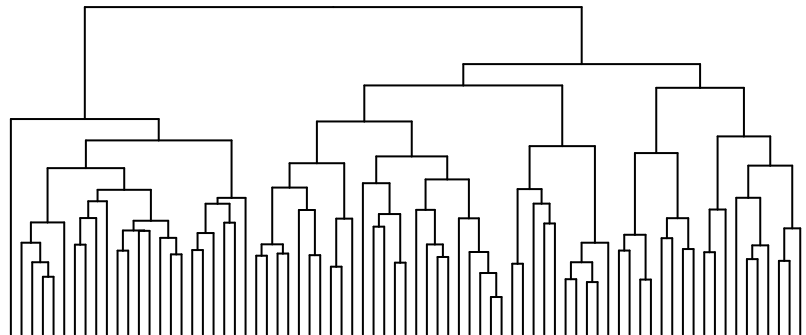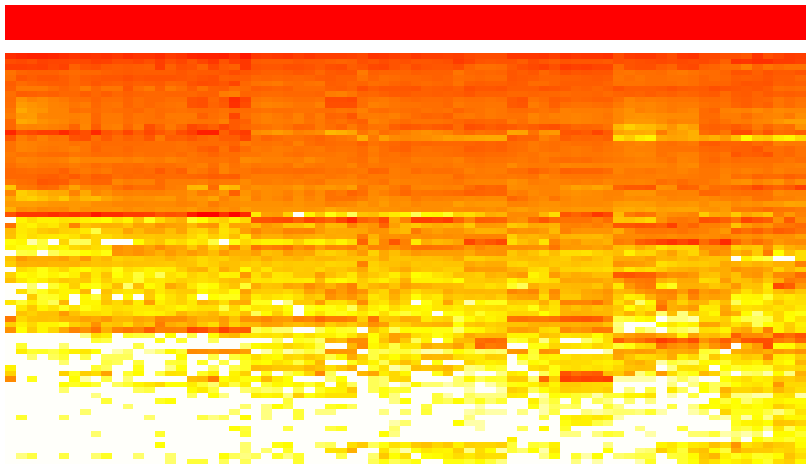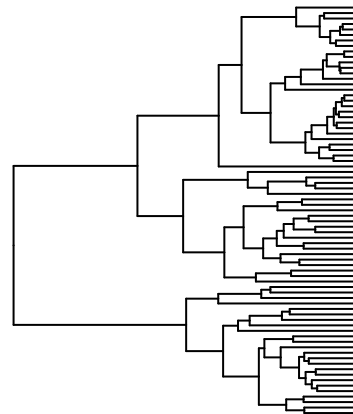[illegible]

Supplement: Supplementary file 1 [file genes-12-00226-s001.zip › Supp.Figures/S2.1.pdf]

## Histogram

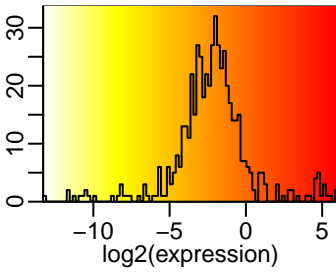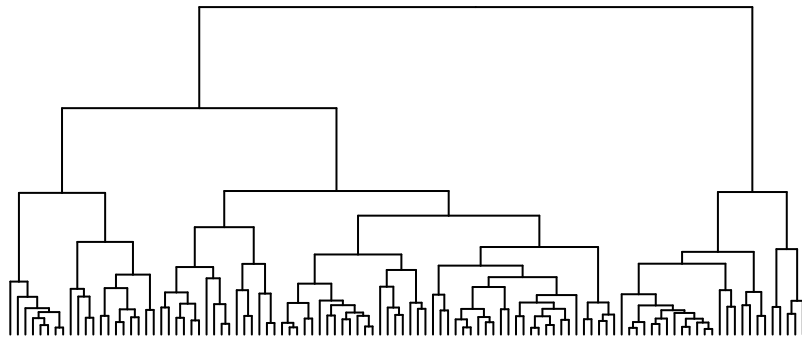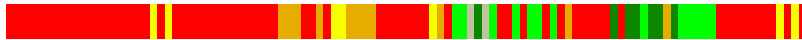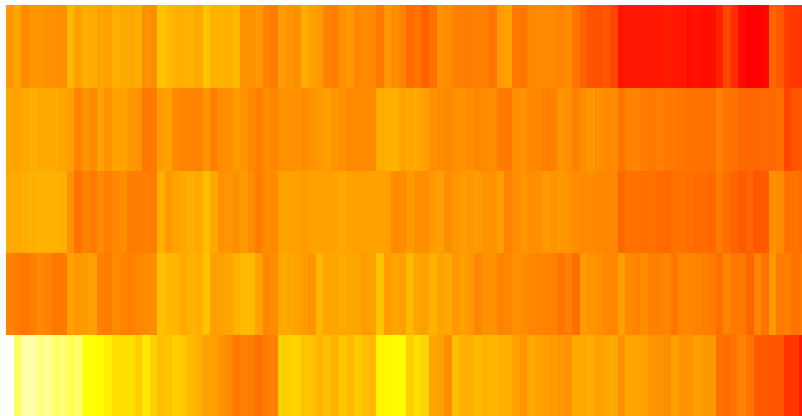

Solyc03g031860

Solyc03g123760

Solyc10g081650

Solyc06g036260

Solyc03g007960

[illegible]

Supplement: Supplementary file 1 [file genes-12-00226-s001.zip › Supp.Figures/S3.2.pdf]

## Histogram

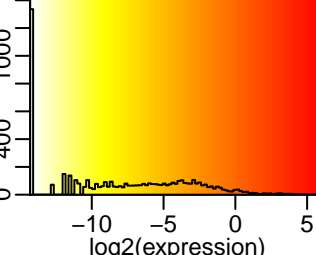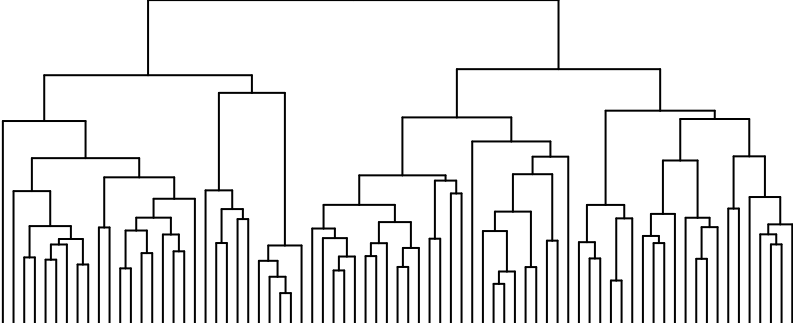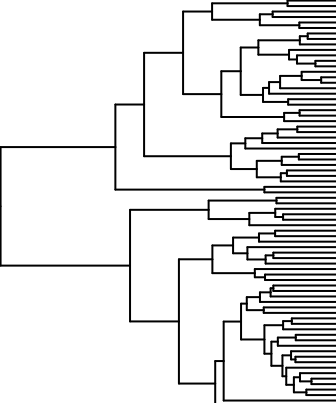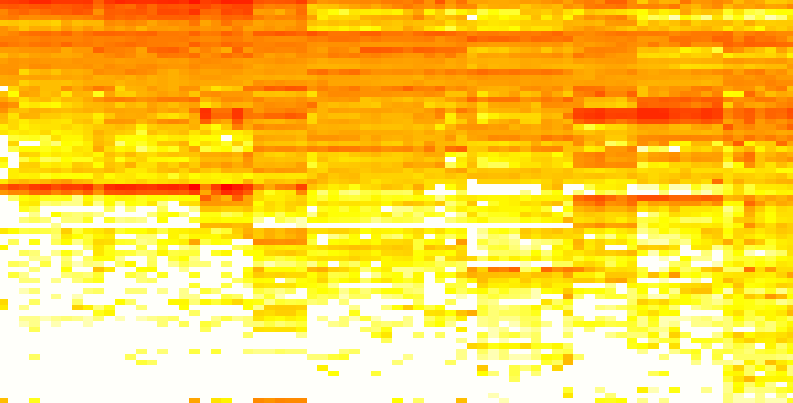[illegible]

Supplement: Supplementary file 1 [file genes-12-00226-s001.zip › Supp.Figures/S4.1.pdf]
